# Supplementary material for: Reduction in Egg Fertility of Aedes albopictus Mosquitoes in Greece Following Releases of Imported Sterile Males
Source: Insects. 2021 Jan 27;12(2):110. doi: 10.3390/insects12020110 (PMC7911890; doi:10.3390/insects12020110)
Supplement: Supplementary file 1 [file insects-12-00110-s001.pdf]

insects

MDPI

Article

Reduction in Egg Fertility of *Aedes Albopictus* Mosquitoes in Greece Following Releases of Imported Sterile Males

Georgios Balatsos [1], Arianna Puggioli [2], Vasileios Karras [1], Ioanna Lytra [1], George Mastronikolos [3], Marco Carrieri [2], Dimitrios P. Papachristos [1], Marco Malfacini [2], Angeliki Stefopoulou [1], Charalampos S. Ioannou [3], Fabrizio Balestrino [2], Jérémy Bouyer [4], Dušan Petrić [5], Igor Pajović [6], Apostolos Kapranas [1], Nikos T. Papadopoulos [3], Panagiotis G. Milonas [1], Romeo Bellini [2] and Antonios Michaelakis [1,*]

[1] Scient. Directorate of Entomology and Agricultural Zoology, Benaki Phytopathological Institute, 14561 Kifissia, Greece; g.balatsos@bpi.gr (G.B.); v.karras@bpi.gr (V.K.); i.lytra@bpi.gr (I.L.); d.papachristos@bpi.gr (D.P.P.); a.stefopoulou@bpi.gr (A.S.); a.kapranas@bpi.gr (A.K.); p.milonas@bpi.gr (P.G.M.)
[2] Centro Agricoltura Ambiente "G. Nicoli", 40014 Crevalcore, Italy; apuggioli@caa.it (A.P.); mcarrieri@caa.it (M.C.); mmalfacini@caa.it (M.M.); fbalestrino@caa.it (F.B.); rbellini@caa.it (R.B.)
[3] Dept. of Agriculture, Crop Production and Rural Environment, University of Thessaly, 38446 Magnisias, Greece; mastronikgeo@ymail.com (G.M.); ioannoubabis@yahoo.com (C.S.I.); nikopap@uth.gr (N.T.P.)
[4] Insect Pest Control Laboratory, Joint FAO/IAEA Programme of Nuclear Techniques in Food and Agriculture, Seibersdorf, A-2444 Vienna, Austria; J.Bouyer@iaea.org
[5] University of Novi Sad, Faculty of Agriculture, 21000 Novi Sad, Serbia; dusan.petric@polj.uns.ac.rs
[6] University of Montenegro, Biotechnical Faculty, 81000 Podgorica, Montenegro; pajovicb.igor@gmail.com
* Correspondence: a.michaelakis@bpi.gr

Citation: Balatsos, G.; Puggioli, A.; Karras, V.; Lytra, I; Mastronikolos, G.; Carrieri, M.; Papachristos, D.P.; Malfacini, M.; Stefopoulou, A.; Ioannou, C.S.; et al. Reduction in Egg Fertility of *Aedes Albopictus* Mosquitoes in Greece Following Releases of Imported Sterile Males. **2021**, *12*, 110. https://doi.org/10.3390/insects12020110

Received: 7 January 2021
Accepted: 22 January 2021
Published: 27 January 2021

Publisher's Note: MDPI stays neutral with regard to jurisdictional claims in published maps and institutional affiliations.

Copyright: © 2021 by the authors. Licensee MDPI, Basel, Switzerland. This article is an open access article distributed under the terms and conditions of the Creative Commons Attribution (CC BY) license (http://creativecommons.org/licenses/by/4.0/).

Supplementary

Table S1. Data for the rainfall and wind speed for the period from week No 37 to No 47 (2018). (based on www.me-teo.gr).

Month	Day	Rain (mm)*	Speed (km/h)*	No of week
September	10	0.0	6.0	
September	11	0.0	4.5	
September	12	0.0	9.0	
September	13	0.0	11.4	37
September	14	0.4	2.7	
September	15	0.0	2.7	
September	16	0.0	5.5	
September	17	0.0	13.5	
September	18	0.0	17.1	
September	19	0.0	15.6	
September	20	0.0	13.2	38
September	21	0.0	11.1	
September	22	0.0	4.8	
September	23	0.0	5.5	
September	24	0.0	6.8	
September	25	0.0	17.9	
September	26	0.0	29.0	
September	27	0.2	24.9	39
September	28	11.8	15.6	
September	29	50.2	11.3	
September	30	29.8	9.8	
October	1	4.8	8.4	
October	2	0.0	6.1	
October	3	0.2	2.4	
October	4	0.0	6.1	40
October	5	0.0	11.9	
October	6	0.0	12.7	
October	7	0.0	5.0	
October	8	0.2	4.3	
October	9	0.0	11.3	
October	10	0.0	11.4	
October	11	0.0	13.4	41
October	12	0.0	10.6	
October	13	0.0	15.3	
October	14	0.0	10.0	
October	15	0.0	7.9	
October	16	0.0	7.2	
October	17	0.0	9.7	
October	18	0.0	7.2	42
October	19	0.0	4.3	
October	20	0.2	4.3	
October	21	0.0	6.6	
October	22	0.2	9.0	43

October	23	0.0	7.4	
October	24	0.0	6.3	
October	25	0.0	11.6	
October	26	0.0	4.8	
October	27	0.0	3.5	
October	28	0.0	6.0	
October	29	0.0	3.5	
October	30	0.2	3.5	
October	31	0.6	3.1	
November	1	0.4	4.5	44
November	2	0.0	10.8	
November	3	0.0	15.0	
November	4	0.0	13.0	
November	5	0.0	9.0	
November	6	0.0	12.7	
November	7	0.0	9.7	
November	8	8.0	1.4	45
November	9	3.0	7.9	
November	10	0.0	5.1	
November	11	0.0	4.0	
November	12	0.0	7.2	
November	13	0.0	16.4	
November	14	0.0	13.5	
November	15	0.0	9.2	46
November	16	0.0	12.6	
November	17	11.4	11.3	
November	18	27.6	5.1	
November	19	0.8	5.5	
November	20	0.4	1.3	
November	21	2.4	8.4	
November	22	0.0	7.2	47
November	23	0.0	10.8	
November	24	0.0	7.2	
November	25	0.0	4.7	

*Average number/day.

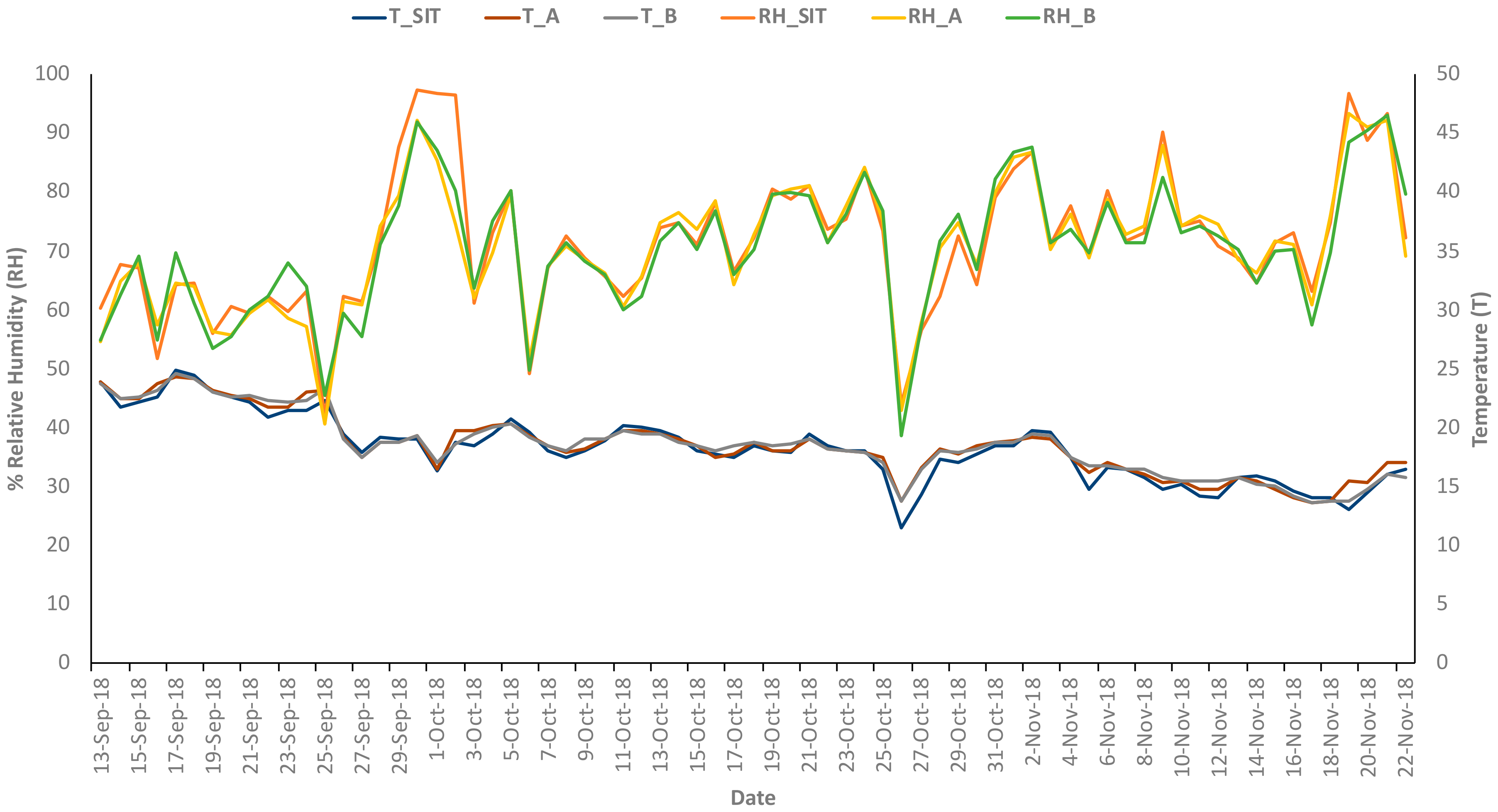

Figure S1. Daily temperature (T) and % relative humidity (RH) for the three areas ("T_SIT" and RH_SIT for SIT area, "T_A" and "RH_A" for Control A and "T_B" and "RH_B" for Control B).

Table S2. Differences in density of eggs per trap per week between the three sites before the start of the release of sterile males. Fixed effects of the generalized poisson linear mixed model fit by maximum likelihood. Control A site used as the reference site (*$p < 0.05$, **$p < 0.01$; ***$p < 0.001$).

	**Estimate**	**SE**	**z value**	**Pr (> |z|)**
(Intercept)	14.7873	0.5055	29.252	$< 2 \times 10^{-16}$ ***
Week	−0.3241	0.0130	−24.934	$< 2 \times 10^{-16}$ ***
Control_B	0.4508	0.3203	1.407	0.1593
SIT	0.7915	0.3193	2.479	0.0132 *

Abbreviation: SE: standard error.

Table S3. Differences in hatch rates between the three sites before the start of the release of sterile males. Fixed effects of the generalized binomial linear mixed model fit by maximum likelihood. Control A site used as the reference site (*$p < 0.05$, **$p < 0.01$; ***$p < 0.001$).

	**Estimate**	**SE**	**z value**	**Pr (> |z|)**
(Intercept)	−26.69813	2.08481	−12.806	$< 2 \times 10^{-16}$ ***
Week	0.70024	0.05957	11.755	$< 2 \times 10^{-16}$ ***
Control_B	−0.01306	0.38006	−0.034	0.973
SIT	−0.06734	0.37352	−0.180	0.857

Abbreviation: SE: standard error.

Table S4. Differences in density of eggs per trap per week between the three sites after the start of the release of sterile males. Fixed effects of the generalized poisson linear mixed model fit by maximum likelihood. Control A site used as the reference site (*$p < 0.05$, **$p < 0.01$; ***$p < 0.001$).

	**Estimate**	**SE**	**z value**	**Pr (> |z|)**
(Intercept)	3.498033	0.303100	11.541	$< 2 \times 10^{-16}$ ***
Week	−0.032980	0.002989	−11.035	$< 2 \times 10^{-16}$ ***
Control_B	1.106567	0.389530	2.841	0.0045 **
SIT	0.442469	0.390695	1.133	0.2574

Abbreviation: SE: standard error.

Table S5. Differences in hatch rates between the three sites after the start of the release of sterile males. Fixed effects of the generalized binomial linear mixed model fit by maximum likelihood. Control A site used as the reference site (*$p < 0.05$, **$p < 0.01$; ***$p < 0.001$).

	**Estimate**	**SE**	**z value**	**Pr (> |z|)**
(Intercept)	9.79811	0.48946	20.018	$< 2 \times 10^{-16}$ ***
Week	−0.17839	0.01095	−16.296	$< 2 \times 10^{-16}$ ***
Control_B	−0.01050	0.22957	−0.046	0.964
SIT	−3.11570	0.23132	−13.469	$< 2 \times 10^{-16}$ ***

Abbreviation: SE: standard error.
